# Preparation of biomass-based hydrogels and their efficient heavy metal removal from aqueous solution

**DOI:** 10.3389/fchem.2022.1054286

**Published:** 2022-12-12

**Authors:** Mingyue Zhang, Yaru Zhou, Fangling Wang, Zeshao Chen, Xu Zhao, Weidong Duan, Guangting Yin, Xinling Yang, Junfeng Li, Quanyu Yin, Mingqin Zhao

**Affiliations:** ^1^ Coll Tobacco Sciences, Flavors and Fragrance Engineering and Technology Research Center Henan, Henan Agriculture University, Zhengzhou, China; ^2^ Shiyan Company, China Tobacco Hubei Industrial Ltd., Shiyan, China; ^3^ China Tobacco Henan Industrial Co Ltd., Zhengzhou, China; ^4^ College of Chemistry, Jilin University, Changchun, China

**Keywords:** heavy metal ions, hydrogel, tobacco straw, adsorption, sulfhydryl modified

## Abstract

In this work, a porous tobacco straw-based polyacrylic acid hydrogel STS-PAA with high adsorption performance was prepared by polymerizing pretreated waste tobacco straw (TS) with acrylic acid/potassium acrylate by UV radiation initiation. The adsorption performance of metal ions was investigated. The effects of different temperatures (25°C, 35°C, and 45°C), adsorption times (1–420 min), pH values (2.0–6.0) and initial concentrations (0.25–4.0 mmol L^−1^) of metal ions on the adsorption amount of heavy metal ions were investigated. The results showed that the hydrogel had a high removal rate of Pb^2+^, Cd^2+^ and Hg^2+^ in aqueous solution. The adsorption of Pb^2+^ was particularly effective. When C_0_ = 4.0 mmol L^−1^, pH = 6, the equilibrium adsorption amount of Pb^2+^, Cd^2+^ and Hg^2+^ reached 1.49 mmol g^−1^, 1.02 mmol L^−1^ and 0.94 mmol g^−1^, respectively. The chemical structure and morphology of the hydrogels were characterized by FT-IR, EDS, SEM and XPS. The Langmuir model fits well with the adsorption system. The kinetic data suggest the adsorption of Pb^2+^, Cd^2+^ and Hg^2+^ follow the pseudo-first-order model. This indicates that STS-PAA adsorption of three heavy metal ions is monolayer physical adsorption. Thermodynamic analysis shows that the adsorption of Pb^2+^, Cd^2+^ and Hg^2+^ by STS-PAA is an endothermic (ΔH>0) entropy increase (ΔS>0) non-spontaneous reaction.

## 1 Introduction

Heavy metals in water cause serious hazards to the ecological environment and human body through bioaccumulation and food chain transfer by aquatic plants and animals (Liu et al., 2018; Abdeldayem, 2020; Li et al., 2020a). How to effectively remove heavy metal ions from water has become the focus of environmental pollution control research (Huang et al., 2019; Mo et al., 2022). The main methods for removing heavy metal ions from aqueous solution are adsorption (Pu et al., 2018), precipitation (Fedje and Strömvall, 2019; Godiya et al., 2019), photodegradation (Dong et al., 2018) and advanced oxidation (e.g. O_3_ and UV/H_2_O_2_) (Kaplan et al., 2020). Among these methods, advanced oxidation and adsorption processes have been widely used in the removal of heavy metal ions. However, the advanced oxidation process may produce harmful byproducts as the target compounds may only change and not be removed from the water, which is not friendly to environmental sustainability. (Wert et al., 2007; Wu et al., 2019).

Adsorption methods are widely used for the removal of heavy metals from water because of their simple design, low operating costs, and ease of treatment. The commonly used adsorbent materials are activated carbon (Prabu et al., 2022), molecular sieve (Yang et al., 2017) and clay (Fei and Hu, 2022). However, conventional adsorbents are limited by pore blockage and hidden surface adsorption sites. The adsorption rate is slow, e.g., the adsorption equilibrium time of activated carbon is up to several hours (Gorzin and Abadi, 2018). Nanosorbents (Pu et al., 2018; Pandey, 2021) exhibit better adsorption equilibrium times. But their complicated preparation process, difficulty in recycling, and easy environmental hazards force us to seek other adsorbents that are low cost, high performance (fast adsorption rate, high efficiency, and good recyclability) and friendly to the environment.

Hydrogels with three-dimensional network structures have been developed for the removal of heavy metal ions from water. These hydrogels are usually polymerized from olefin monomers containing hydrophilic groups, have high permeability, and exhibit excellent adsorption properties for cationic contaminants through electrostatic and hydrogen bonding interactions (Wen et al., 2020). In recent years, biomass-based hydrogels have shown excellent performance in removing pollutants from water. The addition of biomass materials not only improves the adsorption capacity of hydrogels, but also can be degraded by microorganisms and is friendly to the environment (Wong et al., 2021). For example, Sun et al. isolated and extracted lignin from wheat straw and prepared lignin-based hydrogels that demonstrated high adsorption efficiency for copper ions (Sun et al., 2019). Furthermore, it is difficult to achieve rapid, efficient and selective uptake of specific pollutants using a single biomass material. Therefore, surface modification of existing biomass materials with functional groups containing oxygen, nitrogen or sulfhydryl modified groups can help the adsorbents to meet the above requirements (Wen et al., 2017; Liu et al., 2020; Fan et al., 2022). Waste tobacco straw (TS) is a renewable resource with high biomass, friendly to the environment and low price, which makes it an ideal biomass material. These straws contain large amounts of cellulose, hemicellulose and lignin (Syaftika and Matsumura, 2018). It has been shown in the literature that the hydroxyl and amino groups contained in these molecules can be chemically modified to further improve the adsorption capacity of heavy metal ions (Straetz et al., 2019; Miao et al., 2021; Shan et al., 2021). There are few reports related to the removal of heavy metal ions from wastewater by synthetic hydrogels from tobacco straw. Therefore, we innovatively modified tobacco straw by sulfhydryl group to obtain a high adsorption performance of the composite polymer material. This biomass material is cheap and biofriendly.

In this study, modified tobacco straw-based polyacrylic acid hydrogels (STS-PAA) with porous structures were prepared by polymerizing pretreated waste tobacco straw with acrylic acid/potassium acrylate by UV radiation initiation. STS-PAA was used as an adsorbent to remove Pb^2+^, Cd^2+^ and Hg^2+^ from aqueous solutions. The hydrogels were characterized by SEM, FT-IR and XPS. The effects of temperature, adsorption time, pH and initial concentration of metal ions on the adsorption of heavy metal ions were investigated. The adsorption was explored in depth by establishing adsorption kinetics models.

## 2 Experimental

### 2.1 Materials

Acrylic acid (AA) and ammonium persulfate (APS) were purchased from Fuchen Chemical Reagents (Tianjin, China). N, N′-methylenebis (acrylamide) (MBA), benzil dimethyl ketal (BDK), methyl alcohol, sodium hydroxide (NaOH) was purchased from SinopHorm chemical Reagent Co., Ltd. The tobacco straw was obtained from XuChang, Henan province, China, and sieved through 160 mesh steel screen. Pb(NO_3_)_2_, Cd(NO_3_)_2_·4H_2_O and Hg(NO_3_)_2_·H_2_O by SinopHorm chemical Reagent Co. Ltd. These reagents were of analytical grade and prepared with deionized water.

### 2.2 Sample preparation

#### 2.2.1 Pretreatment of tobacco straw

Firstly, 50 g of tobacco straw was dispersed into NaOH solution (10wt%) and stirred magnetically for 2 h at 95°C. After the solution returned to normal temperature, NaClO/H_2_O_2_ (volume ratio was 3: 4) mixed solution were added to the above solution and soak for 8 h. Afterwards, the solution was filtered out and was repeatedly washed with pure water to pH = 7. Finally, the wet tobacco straw was dried in a drying oven at 60°C for 24 h to obtain a dry pretreatment of tobacco straw.

#### 2.2.2 Synthesis of sulfhydryl modified tobacco straw

The reactions were carried out in a 1000.00 ml round-bottom flask fitted with a reflux condenser and oil-bath under atmospheric pressure. The stirring speed was 600 rpm. Firstly, 30.00 g pre-treated tobacco straw, 0.75 g Na_2_S·9H_2_O, 75.00 ml dimethyl formamide and 150.00 ml sulfhydryl acetic acid were added to the flask. The mixture reacted for 150 min at 120°C. After the solution returned to normal temperature, 180.00 g Na_2_S·9H_2_O and 750.00 ml absolute ethanol solution were added to the above solution and stirred magnetically for 60 min. Finally, the solution was filtered out and the sample is repeatedly washed with pure water to pH = 7. Then it was dried in a vacuum drying oven at 60°C to a constant weight, and then crushed for subsequent experiments to obtain STS.

#### 2.2.3 Synthesis of sulfhydryl modified tobacco straw-based polyacrylic acid

First, a certain amount of KOH solution is neutralized with acrylic acid under ice-water bath conditions. After the solution returned to normal temperature, STS was added and sonicated for 30 min to make it uniformly mixed. Then, the cross-linking agent MBA, the thermal initiator APS and the photoinitiator BDK solution were added to the above solution. And ultrasonic treatment was performed for 1 min. Then the whole system was irradiated with UV light (250 W, *λ* = 365 nm) for 3 min to obtain STS-PAA. At long last, the synthesized hydrogel sample was soaked and washed with absolute ethanol overnight to remove excess unreacted monomer. The sample was dried in a drying oven at 60°C to a constant weight, and then crushed for subsequent experiments. PAA was synthesized by the same method without adding tobacco straw.

### 2.3 Adsorption performance test

Firstly, a specified amount of dried STS-PAA (0.03 g) and prepared heavy metal ion (Pb^2+^, Cd^2+^, Hg^2+^) solution (35 ml) was taken into a 50 ml centrifuge tube and stirred using a SHZ-82A thermostatic water bath shaker (200 rpm) for certain time (t min). Secondly, the tube was taken out and centrifuged at 8000 rpm for 5 min. Then the supernatant was sucked by a syringe. Finally, through the adsorption capacity of adsorbents measured by ICP-MS. The adsorption capacity of samples at time t (q_t_, mmol g^−1^) and the equilibrium adsorption capacity (q_e_, mmol g^−1^) were calculated according to Eqs 1, 2:

$$q_{t}=\frac{\left(C_{0}-C_{t}\right)V}{m}$$ (1)

$$q_{e}=\frac{\left(C_{0}-C_{e}\right)V}{m}$$ (2)

Where C_0_ represents the initial concentration of metal ions (mmol L^−1^). C_t_ speaks to the concentration of the solution at time t (mmol L^−1^). C_e_ represents the equilibrium concentration (mmol L^−1^). V (ml) is the volume of the metal ions solution and m (g) was the dose of the adsorbent.

#### 2.3.1 Effect of solution pH on adsorption capacity

Firstly, prepare 0.10 mol L^−1^ HCl and NaOH solution to modify the pH range of the Pb^2+^, Cd^2+^ and Hg^2+^ solutions to 2.0–6.0. Then, 0.03 g STS-PAA was added to Pb^2+^, Cd^2+^ and Hg^2+^ solutions with different pH values at 25°C. (V = 35 ml, C_0_ = 2.5 mmol L^−1^), placed in a constant temperature shaker at 25°C to shake for 120 min (200 rmp).

#### 2.3.2 Effect of temperature on adsorption capacity

0.03 g of STS-PAA was added to 35 ml Pb^2+^, Cd^2+^ and Hg^2+^ solutions with a concentration of 2.00 mmol L^−1^ and pH = 6. The solution was placed in a constant temperature shaker at 25°C, 35°C and 45°C, respectively, for 120 min.

#### 2.3.3 Effect of time on adsorption capacity

At 25°C, 0.03 g STS-PAA was put into 35 ml, initial concentration 2.5 mmol L^−1^, pH = 6, Pb^2+^, Cd^2+^ and Hg^2+^ solution, respectively. While the shaking time was 1, 3, 5, 7, 9, 10, 20, 30, 40, 50, 60, 80, 100, 120, 150, 180, 210, 240, 270, 300, 330,360, 390, 420 min, respectively.

#### 2.3.4 Effect of metal ions concentration on adsorption capacity

First, 5 mmol L^−1^ Pb^2+^, Cd^2+^ and Hg^2+^ solutions were prepared with Pb(NO_3_)_2_, Cd(NO_3_)_2_·4H_2_O and Hg(NO_3_)_2_·H_2_O and distilled water. Then, the solution with concentration of 0.25, 0.50, 1.00, 1.50, 2.00, 2.50 mmol L^−1^ was obtained by dilution, respectively. At 25°C, pH = 6, 0.03 g STS-PAA was put into the solution, which was shaken at a rate of 200 rpm for 120 min.

### 2.4 Adsorption behavior studies

#### 2.4.1 Study on adsorption kinetics

The study of adsorption kinetics is mainly to explore the adsorption mechanism. To better study the adsorption process, pseudo-first-order kinetic model, pseudo-second-order kinetic model and intra-particle diffusion model were used. The equations are as follows:

Pseudo-first-order kinetic model:

$$q_{t}=q_{e}(1-e^{-k_{1}t})$$ (3)

Pseudo-second-order kinetic model:

$$q_{t}=\frac{q_{e}^{2}k_{2}t}{1+q_{e}k_{2}t}$$ (4)

Intra-particle diffusion model:

$$q_{t}=K_{p}t^{0.5}+C$$ (5)

In the equation, q_t_ (mmol g^−1^) is the adsorption amount at t (min). qe (mmol g^−1^) is the adsorption amount when the adsorption reaches equilibrium. k_1_, k_2_ and k_p_ are the rate constants of the pseudo-first-order kinetic model, pseudo-second-order kinetic mode, and intra-particle diffusion model. C is a constant related to the boundary layer and thickness around the adsorbent.

#### 2.4.2 Study on adsorption isotherm

Adsorption isotherms provide a favorable basis for studying the chemical interactions between homogeneous and heterogeneous adsorbents. Further analysis of the adsorption of Pb^2+^, Cd^2+^, and Hg^2+^ to understand the adsorption mechanism in depth, the distribution of solute in the solid-liquid phase during adsorption can be studied by using different adsorption isotherms. The four adsorption isotherm model equations are as follows:

Langmuir isotherm model:

$$q_{e}=\frac{q_{m,l}K_{L}C_{e}}{1+K_{L}C_{e}}$$ (6)

Freundlich isotherm model:

$$q_{e}=K_{F}C_{e}^{\frac{1}{n}}$$ (7)

Temkin isotherm model:

$$q_{e}=B\times \ln\left(AC_{e}\right)$$ (8)

Dubinin-Radushkevich isotherm model:

$$q_{e}=q_{m,D-R}e^{-\beta \varepsilon^{2}}$$ (9)

$$\varepsilon =RTln\left(1+\frac{1}{C_{e}}\right)$$ (10)

where q_e_ is the amount of adsorbed molecules at equilibrium (mmol∙g^−1^), q_m,l_ is the maximum adsorption capacity (mmol∙g^−1^), q_m,D-R_ represent the maximum theoretical adsorption capacity of D-R models (mmol∙g^−1^), C_e_ is the equilibrium concentration (mmol∙L^−1^), n is the adsorption strength, K_L_ (L·mmol^−1^) is the Langmuir constant (L∙mmol^−1^), K_F_ (L∙mmol^−1^) is the Freundlich constant (L∙mmol^−1^), A (mg L^−1^) and B represent Temkin model constants associated with binding energy and adsorption heat, respectively; ε is the adsorption potential; β(mol^2^ KJ^−2^) is the adsorption constant related to the adsorption energy; R (J K^−1^ mol−1) is the general constant of gas; T (K) is the reaction temperature.

#### 2.4.3 Study on thermodynamic study

To determine the thermodynamic behavior of the adsorption manner (endothermic or exothermic, spontaneous, or non-spontaneous), thermodynamic parameters such as entropy ( ∆ S, J/K.mol), enthalpy ( ∆ H, kJ/mol), and Gibbs free energy ( ∆ G, kJ/mol) were calculated by following equations.

$$∆G=-RTlnK_{D}$$ (11)

$$K_{D}=\frac{q_{e}}{C_{e}}$$ (12)

∆G=∆H−T∆S (13)

$$\ln K_{D}=\frac{∆S}{R}-\frac{∆H}{RT}$$ (14)

where R is the gas constant (8.314 J/mol K), K_D_ equal to q_e_/C_e_ is adsorption affinity, and T is the temperature in Kelvin. The constants ΔH and ΔS were calculated by the slope and intercept.

### 2.5 Characterization

The microscopic surfaces of STS-PAA were observed by scanning electron microscopy (SEM, Zeiss Gemini 300) and energy dispersive spectrometry (EDS, bruker, Germany). The chemical structures of STS-PAA before and after adsorption of heavy metal ions were studied by a Fourier transform infrared spectroscope (FT-IR, Nicolet iS10). Thermogravimetric analysis (TGA) was performed by using an STA 449C integrated thermal analyzer (Netzsch, Germany) at 10°C–600°C with the rate of 10°C min^−1^ under nitrogen flow. X-ray photoelectron spectroscopy (XPS, Thermo Kalpha) was used to analyze the chemical state of the sample surface before and after adsorption. ICP-MS (Agilent, G8421A) measured the metal ion concentration.

## 3 Results and discussion

### 3.1 Synthesis and adsorption scheme for sulfhydryl modified tobacco straw-based polyacrylic acid

The synthesis mechanism of STS-PAA is divided into three processes. Firstly, chain initiation: the initiator generates initial radicals under UV irradiation. The initial radical further interacts with cellulose in tobacco straw and monomer to generate hydroxyl radicals and monomer radicals. Secondly, chain growth: the monomer radicals interact with the monomer and cellulose. The cross-linker participates in the reaction to form a three-dimensional network structure. Thirdly, chain termination: as the reaction proceeds, the monomer content continues to decrease until the reaction is complete, and the polymerization is finished.

**FIGURE 1:**
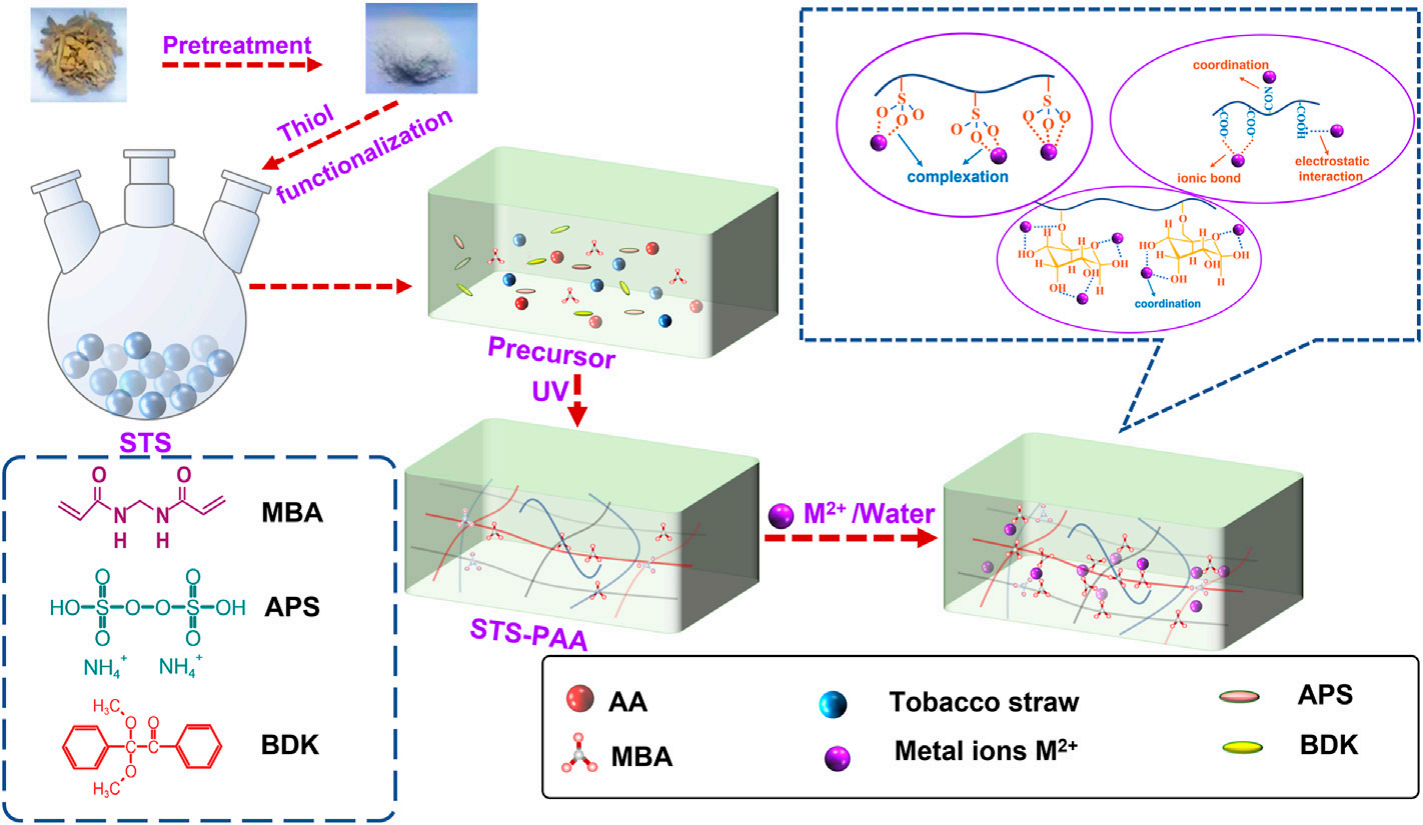
Synthesis and adsorption scheme for STS-PAA.

The adsorption of heavy metal ions by STS-PAA is dominated by chemisorption and supplemented by physical adsorption. It mainly includes ion exchange between -COOK on the monomer chain and heavy metal ions, coordination between -OH and heavy metal ions, interaction between–COOH and heavy metal ions, complexation between -SO_3_ and heavy metal ions and coordination between–CONH and heavy metal ions. The details are shown in Figure 1.

### 3.2 Characterization of sulfhydryl modified tobacco straw-based polyacrylic acid

#### 3.2.1 Scanning electron microscopy analysis

**FIGURE 2:**
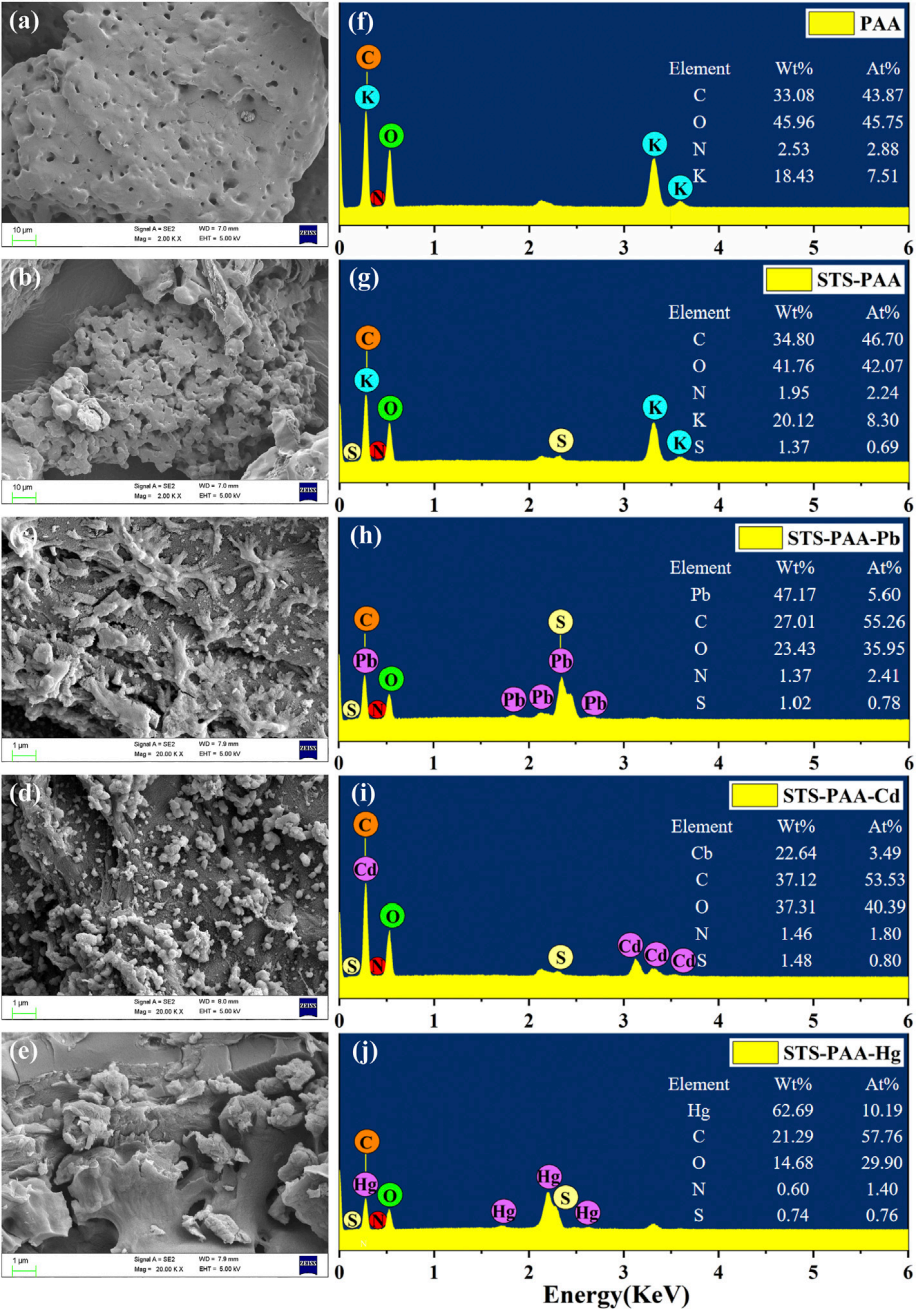
SEM images of **(A)** PAA, **(B)** STS-PAA, **(C)** STS-PAA-Pb, **(D)** STS-PAA-Cd and **(E)** STS-PAA-Hg, EDS of **(F)** PAA, **(G)** STS-PAA, **(H)** STS-PAA-Pb, **(I)** STS-PAA-Cd and **(J)** STS-PAA-Hg.

The microscopic morphology of the sample was characterized by SEM. From Figure 2A, uneven pores can be clearly observed on the surface of PAA. This can be caused by evaporation of water during the drying process. Modified hydrogel (Figure 2B) surface is more irregular, rough and has open porous structure. The porous structure of hydrogel could provide important prerequisite for the adsorption of metal ions and easy collectability.

Figures 2C,D,E are the surface of hydrogel after adsorbed Pb^2+^, Cd^2+^ and Hg^2+^ ions. Their surface is covered by particles of different particle sizes, indicating the successful adsorption of heavy metal ions. The atomic species and elemental percentages in the samples were analyzed by EDS and the results also showed that the adsorbed hydrogel contained heavy metal ions. From Figures 2F,G, it can be seen that element S was not detected in PAA, but 1.37 wt% S was detected in STS-PAA after adding modified tobacco straw. This indicates that the sulfhydryl modification of tobacco straw has been successfully realized and the modified tobacco straw participated in the synthesis of the hydrogel. After adsorption of Pb^2+^, Cd^2+^ and Hg^2+^ ions, element Pb, Cd and Hg were detected (Figure 2H–J). Their levels were 47.17 wt%, 22.64 wt% and 62.69 wt%, respectively. This visually demonstrated the successful adsorption of Pb^2+^, Cd^2+^ and Hg^2+^ ions by the hydrogel.

#### 3.2.2 Fourier transform infrared spectroscope analysis

**FIGURE 3:**
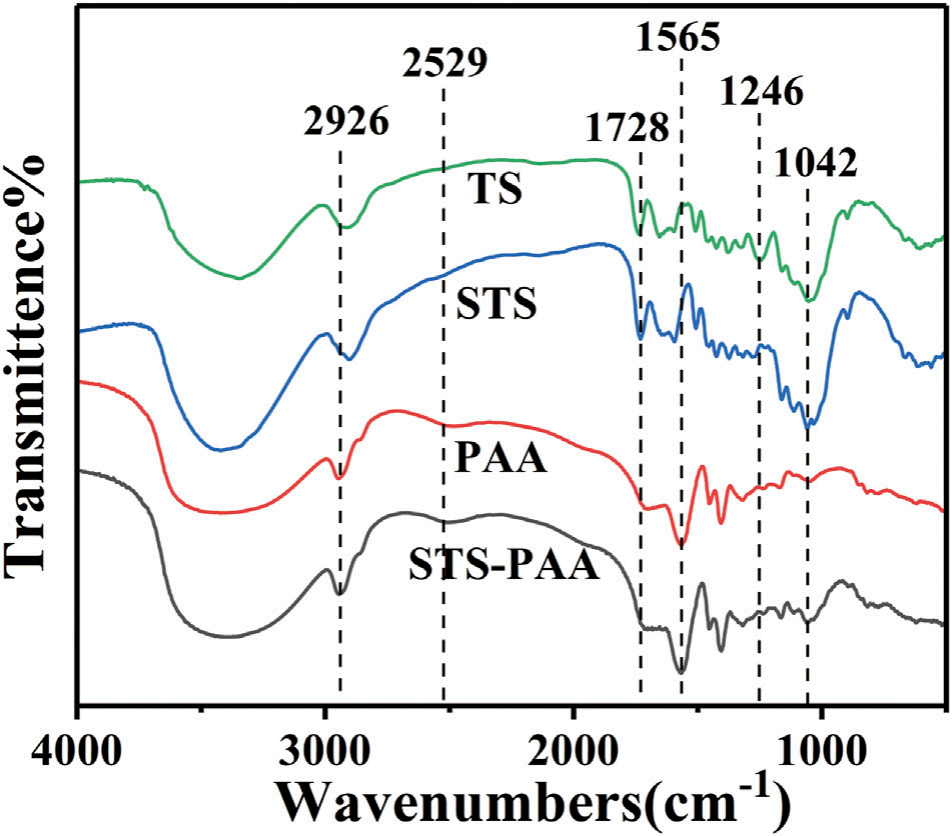
FT-IR spectra of TS, STS, PAA and STS-PAA.

The infrared spectra of TS, STS, PAA and STS-PAA are shown in Figure 3. From the spectra of TS and STS, it can be seen that the adsorption peaks at 3419 cm^−1^, 2926 cm^−1^, 1246 cm^−1^ and 1042 cm^−1^ are attributed to O-H (Mukherjee et al., 2018), C-H (Ahmad and Mirza, 2018), C-O-C (Rodrigues Filho et al., 2008) and C-O stretching vibrations (Emam and Shaheen, 2022), which are characteristic adsorption peaks of cellulose. It proves that TS and STS contains cellulose. The characteristic adsorption peaks of STS belonging to cellulose are slightly different from those of TS because sulfhydryl groups are introduced into TS. The peak located at 2529 cm^−1^ belongs to the sulfhydryl group, which proves the successful introduction of the sulfhydryl group. For the polymer PAA, the peaks at 2926 cm^−1^, 1694 cm^−1^, 1565 cm^−1^ and 1403 cm^−1^ are derived from the stretching vibration of C-H, C-N, C=O and amide II (from the cross-linker MBA) co-action peak, -COO-symmetric stretching vibration. In contrast to STS, STS-PAA shows new adsorption peaks at positions 1316 cm^−1^ (C-O-C) and 1056 cm^−1^ (C-O), which are characteristic peaks of the cellulose structure. It indicates that the cellulose in STS is involved in the polymerization reaction.

#### 3.2.3 X-ray photoelectron spectroscopy analysis

**FIGURE 4:**
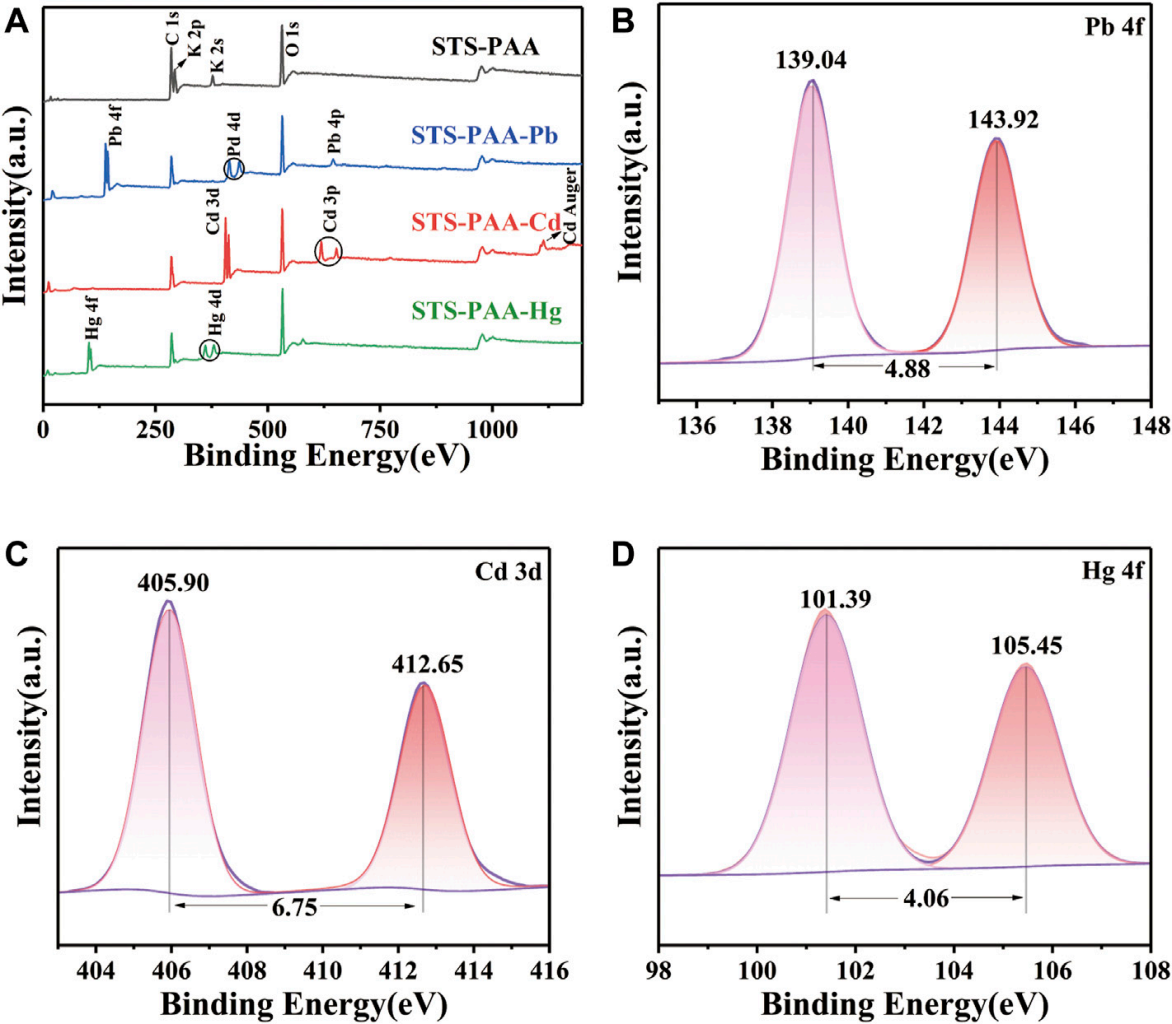
XPS spectra of **(A)** STS-PAA before and after adsorption metal ions, **(B)** Pb 4f of STS-PAA-Pb, **(C)** Cd 3d STS-PAA-Cd and **(D)** Hg 4f of STS-PAA-Hg.

STS-PAA before and after adsorption of Pb^2+^, Cd^2+^ and Hg^2+^ ions were characterized by XPS in order to analyze the surface composition of STS-PAA and the adsorption mechanism. As shown in Figure 4A, C1s, O1s, K 2p and K 2s were observed in STS-PAA. And the K peaks disappeared after the adsorption of metal ions. Correspondingly, after adsorption of Pb, Pb 4f peaks appeared at 139.04 eV and Pb 4d peak at 413.92 eV and Pb 4p peak at 645.01 eV. It proves that STS-PAA successfully adsorbed Pb and exchanged ions with -COOK in STS-PAA to form -(COO)_2_Pb. After adsorption of Cd^2+^ ions, new Cd 3d and Cd 3p peaks appeared at 404.92 eV and 617.93 eV. It proves that STS-PAA successfully adsorbed Cd, forming -(COO)_2_Cd. After adsorption of Hg^2+^ ions, new Hg 4f and Hg 4d peaks appeared at 104.46 eV and 378.17 eV. It proves that Hg^2+^ replaced K^+^ on the polymer chain and Hg^2+^ was successfully adsorbed on the polymer chain.

The fitted spectra of Pb 4f for STS-PAA-Pb, Cd 3d for STS-PAA-Cd and Hg 4f of STS-PAA-Hg are shown in Figures 4B,C,D. The peaks of Pb 4f5/2 and Pb 4f7/2 appear at 143.92 eV and 139.04 eV, respectively. The difference between the two peaks is 4.88 eV, which is in general agreement with the standard value of 4.80 eV. The peaks of Cd 3d5/2 and Cd 3d3/2 occurred at 405.90 eV and 412.65 eV, respectively. The difference between the two peaks was 6.75 eV, which was in general agreement with the standard value of 6.70 eV. The peaks of Hg 4f7/2 and Hg 4f5/2 appear at 101.39 eV and 105.45 eV, respectively. And the difference between the two peaks is 4.06 eV, which is in general agreement with the standard value of 4.00 eV.

**FIGURE 5:**
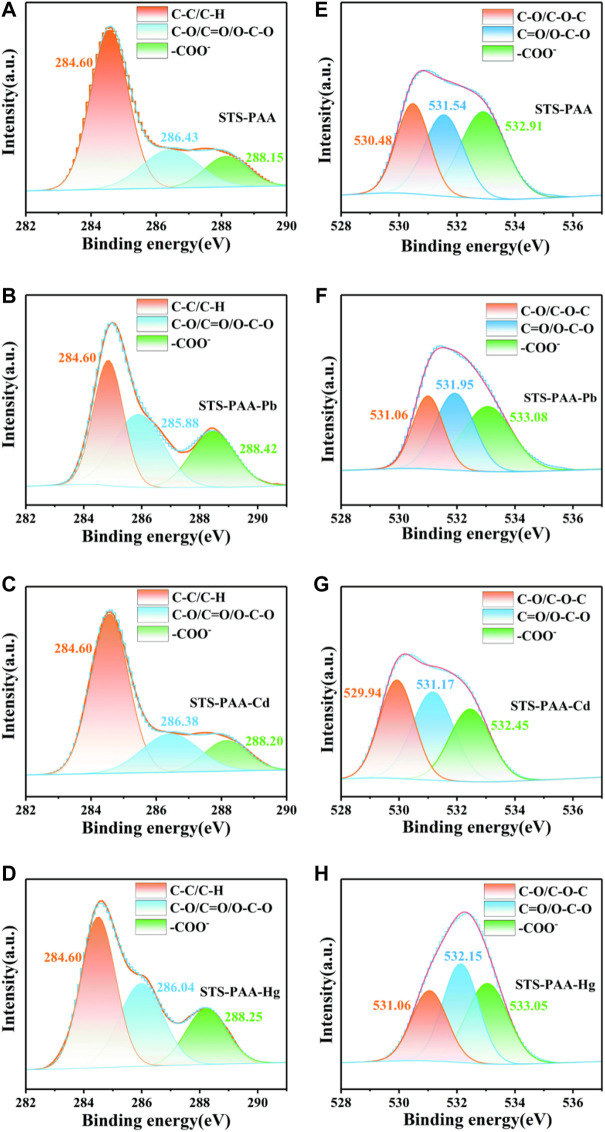
XPS spectra of **(A–D)** C1s, **(E–H)** O1s peaks for STS-PAA, STS-PAA-Pb, STS-PAA-Cd and STS-PAA-Hg.

Using a Gaussian fit, the O1s and C1s spectra were convolved into three peaks (Figure 5). 284.60 eV of the C1s belonged to C-C and C-H, 286.43 eV to C-O, O-C-O and C=O, 288.15 eV to -COO^-^ (Song et al., 2021) (Figure 5A). After the adsorption of heavy metal ions, the peaks at 286.43 eV were all shifted to lower BE, which due to the interaction of Pb^2+^, Cd^2+^ and Hg^2+^ with C-OH. The peaks at 288.15 eV were all shifted to higher BE. It due to the binding of metal ions with -COO^-^ to generate - (COO)_2_Pb, -(COO)_2_Cd and -(COO)_2_Hg. 530.48 eV belonged to C-OH and C-O-C, 531.54 eV belonged to C=O and O-C-O, and 532.91 eV belonged to -COO^-^ in O1s (Figure 5E). After adsorption Pb^2+^ and Hg^2+^, all three peaks move to higher BE. It was because the interaction of Pb and Hg with oxygen atoms, which reduced their electron density. Similarly, Pb^2+^, Cd^2+^ and Hg^2+^ could be found in the XPS patterns after the adsorption of STS-PAA, proving that the heavy metal ions were successfully adsorbed.

#### 3.2.4 Thermogravimetric analysis

**FIGURE 6:**
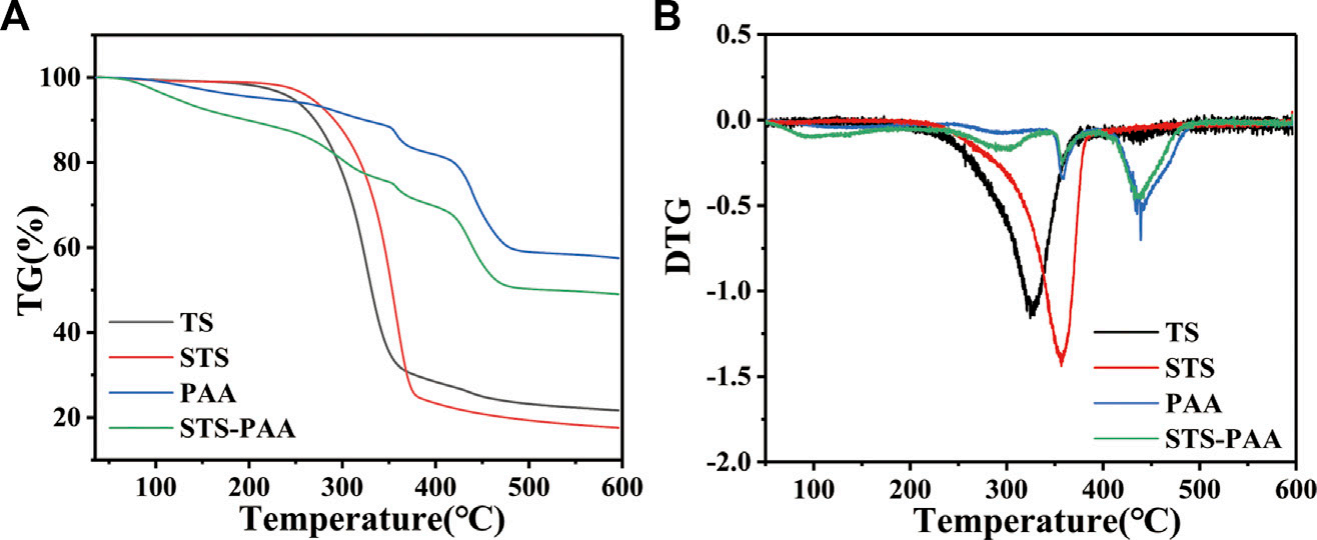
**(A)** TG and **(B)** DTG curves of TS, STS, PAA and STS-PAA.

Through TGA analysis, we can further explore the thermal stability of the hydrogel. As shown in the Figure 6, the total weight loss of TS, STS, PAA and STS-PAA were 78.26%, 82.50%, 42.63% and 50.84%, respectively. The DTG curves showed that the pyrolysis process of PAA consisted of four stages: 72.94°C–153.04°C, 247.12°C–334.42°C, 344.11°C–386.70°C and 405.51°C–490.33 °C, with weight losses of 3.78%, 5.21%, 5.27%, and 22.34%, respectively. For STS-PAA, the first stage of weight loss occurred at 54.38°C–170.38°C with 8.97% weight loss, which was caused by the evaporation of adsorbed water from the hydrogel. The second stage occurred at 234.08°C–345.18°C with a weight loss of 13.02%, which was related to the decomposition of branched chains and the breakage of weak chemical bonds such as hydroxyl groups, esters, and C-O-C. More precisely, it is the decomposition of cellulose chains and the reaction between polymer backbones to remove H_2_O and CO_2_ molecules such as adjacent carboxyl groups to form anhydride dehydration and decarboxylation between carboxyl groups. Because of the faster decomposition rate, the degradation rate of STS-PAA is greater than that of PAA at this stage after the reaction with STS. The weight loss occurring at 348.00°C–383.12°C was 4.47%, due to the further oxidation and breakage of the cross-linked network structure. The decomposition of residual organic matter resulted in a weight loss of 17.74% in the last stage (409.03°C–483.21°C). The final residual amount was 50.54%, which was mainly residual inorganic salts and little organic matter. In addition, the addition of STS led to the residual amount of STS-PAA was less than that of PAA (58.86%) and more than that of STS (24.09%).

#### 3.3.1 Effect of pH on adsorption performance

**FIGURE 7:**
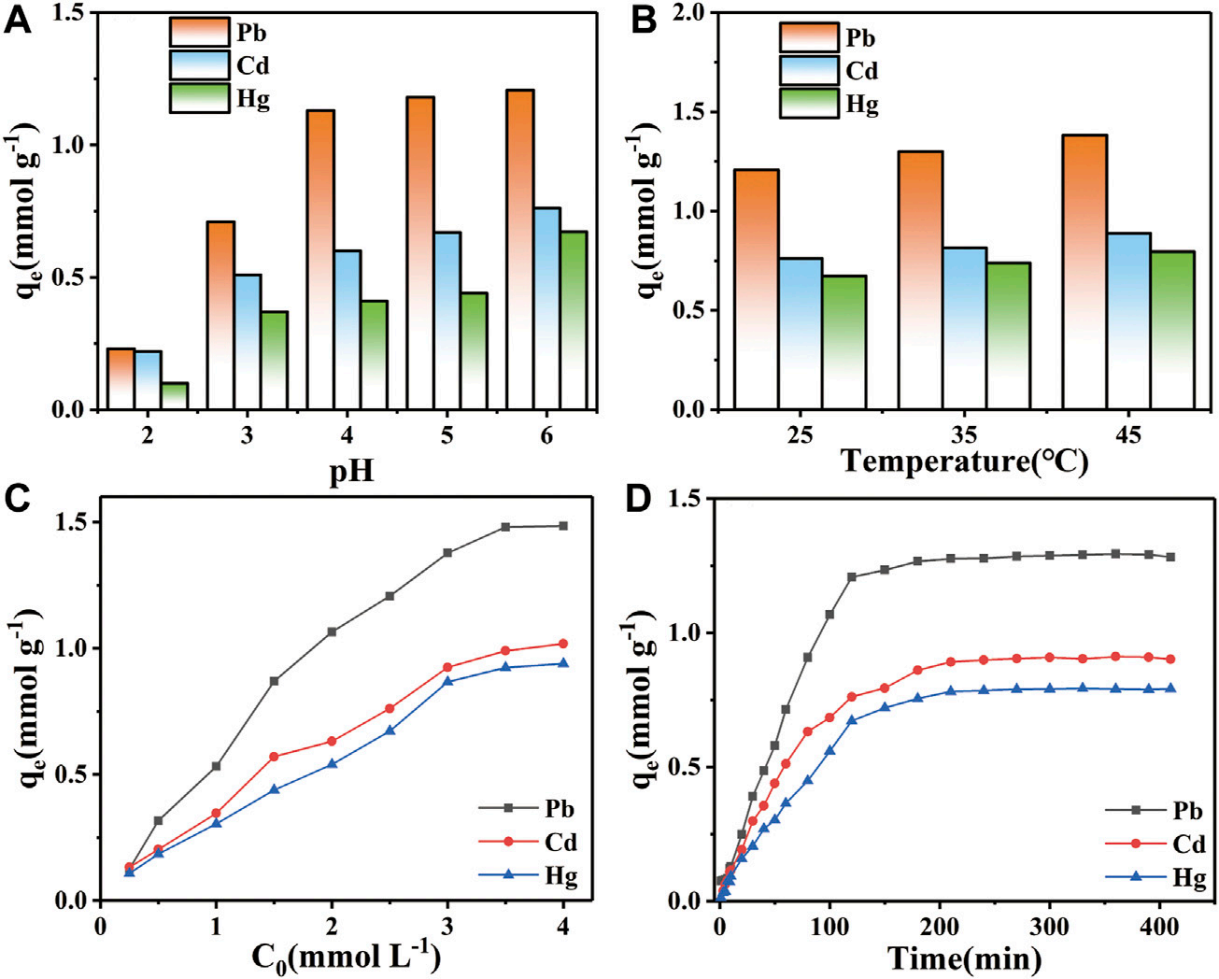
Effect of **(A)** pH value, **(B)** temperature, **(C)** adsorption time and **(D)** initial concentration on Pb^2+^, Cd^2+^ and Hg^2+^ ions adsorption.

As shown in Figure 7A, the equilibrium adsorption of Pb^2+^ were 0.23, 0.71, 1.13, 1.18 and 1.21 mmol g^−1^ at pH 2, 3, 4, 5 and 6, respectively. The equilibrium adsorption of Cd^2+^ were 0.22, 0.51, 0.60, 0.67 and 0.76 mmol g^−1^, respectively. The equilibrium adsorption of Hg^2+^ were 0.10, 0.37, 0.41, 0.44 and 0.67 mmol g^−1^, respectively. The equilibrium adsorption of STS-PAA for the three heavy metal ions showed an increasing trend with the increase of pH value. Meanwhile, the equilibrium adsorption of the three heavy metal ions were Pb^2+^ > Cd^2+^ > Hg^2+^ regardless of the pH values. This can be explained by the fact that the surface functional groups of STS-PAA were occupied by protons in a strongly acidic environment, which weakened the chelating effect of the adsorbent with heavy metal ions. At the same time, the positive charge on the adsorbent surface interacted with heavy metal ions by electrostatic repulsion, thus reducing the adsorption capacity. With the increase of pH, the functional groups on the surface of STS-PAA gradually deprotonated and the surface charge changed from positive to negative, thus improving the interaction between the functional groups and heavy metal ions.

#### 3.3.2 Effect of temperature on adsorption performance

Figure 7B shows the effect of temperature on the adsorption of STS-PAA for Pb^2+^, Cd^2+^ and Hg^2+^ at 25°C, 35°C and 45°C. At 25°C, the equilibrium adsorption of STS-PAA for Pb^2+^, Cd^2+^ and Hg^2+^ was 1.21, 0.76 and 0.67 mmol g^−1^, respectively. At 35°C, the equilibrium adsorption of STS-PAA for Pb^2+^, Cd^2+^ and Hg^2+^ was 1.30, 0.81 and 0.74 mmol g^−1^, respectively. At 45°C, the equilibrium adsorption of STS-PAA for Pb^2+^, Cd^2+^ and Hg^2+^ was 1.38, 0.89, and 0.79 mmol g^−1^, respectively. The equilibrium adsorption of STS-PAA for heavy metal ions showed an increasing trend with increasing temperature. The maximum adsorption amounts of STS-PAA for Pb^2+^and Cd^2+^ were observed at 45°C. This indicates that the ion adsorption reaction is a heat absorption reaction, and the high temperature is favorable for the particle adsorption.

#### 3.3.3 Effect of concentration on adsorption performance

Figure 7C shows that the adsorption capacity first increases and then reaches equilibrium as the initial concentration of heavy metal ions increases. This can be explained by the fact that when the pollutant concentration increases, the concentration difference between the two sides of the adsorbent surface increases, which facilitates the binding of the pollutant to the adsorption site. However, the number of adsorption sites of the adsorbent is limited. When the pollutant concentration reaches a certain value, all the adsorption sites of the adsorbent will be occupied, thus reaching the adsorption equilibrium.

#### 3.3.4 Effect of time on adsorption performance

Figure 7D shows that the adsorption of the three metal ions are basically the same in relation to time. With the change of time, the three pollutants gradually reached adsorption equilibrium from rapid adsorption. This can be explained by the fact that the early surface of the STS-PAA adsorbent is rich in functional groups, which can provide sufficient adsorption sites. In addition, the surface of the STS-PAA adsorbent had more negative charges, which provided a strong electrostatic attraction for the adsorbed pollutants. As the number of adsorption sites decreases in the later stages, the surface of STS-PAA is gradually occupied by the pollutants. The adsorption starts to reach saturation and the adsorption rate gradually decreases and finally reaches equilibrium. In addition, it can be seen from Table 1 that the metal ion adsorption capacity of STS-PAA have significantly higher than other materials. Adsorption equilibrium time also has advantages compared to other materials.

**TABLE 1:** Metal ions adsorption of STS-PAA in the present work in comparison with recently reported results.

Adsorption capacity/mmol g^−1^					
Absorbents	Pb^2+^	Cd^2+^	Hg^2+^	Time	References
SMAHS		1.00		20 h	Satya et al. (2021)
BS-N62		0.04		2 h	Landin-Sandoval et al. (2020)
HFO-BC		0.27		24 h	Li et al. (2020b)
*Pseudomonas* sp.		0.83		120 min	Xu et al. (2020)
375					
Co-Fe_2_O_3_/Ni-Fe_2_	0.66/0.47			24 h	Chen et al. (2019)
O_3_					
modified chitosan	0.56			240 min	Badawi et al. (2017)
Biopolymer					
PVA/CNF	0.53		0.78	3 days	Zheng et al. (2014)
aerogel					
MFC-N	0.49		0.3	60 min	Huang et al. (2018)
Thiol functionalized	1.04			120 min	Ke et al. (2017)
Fe_3_O_4_@MOF					
Fe_3_O_4_–SO_3_H	0.53	0.72		60 min	Chen et al. (2017)
MNPs					
STS-PAA	1.49	1.02	0.94	100 min	this study

### 3.4 Study on adsorption behavior

#### 3.4.1 Study on adsorption kinetics

**FIGURE 8:**
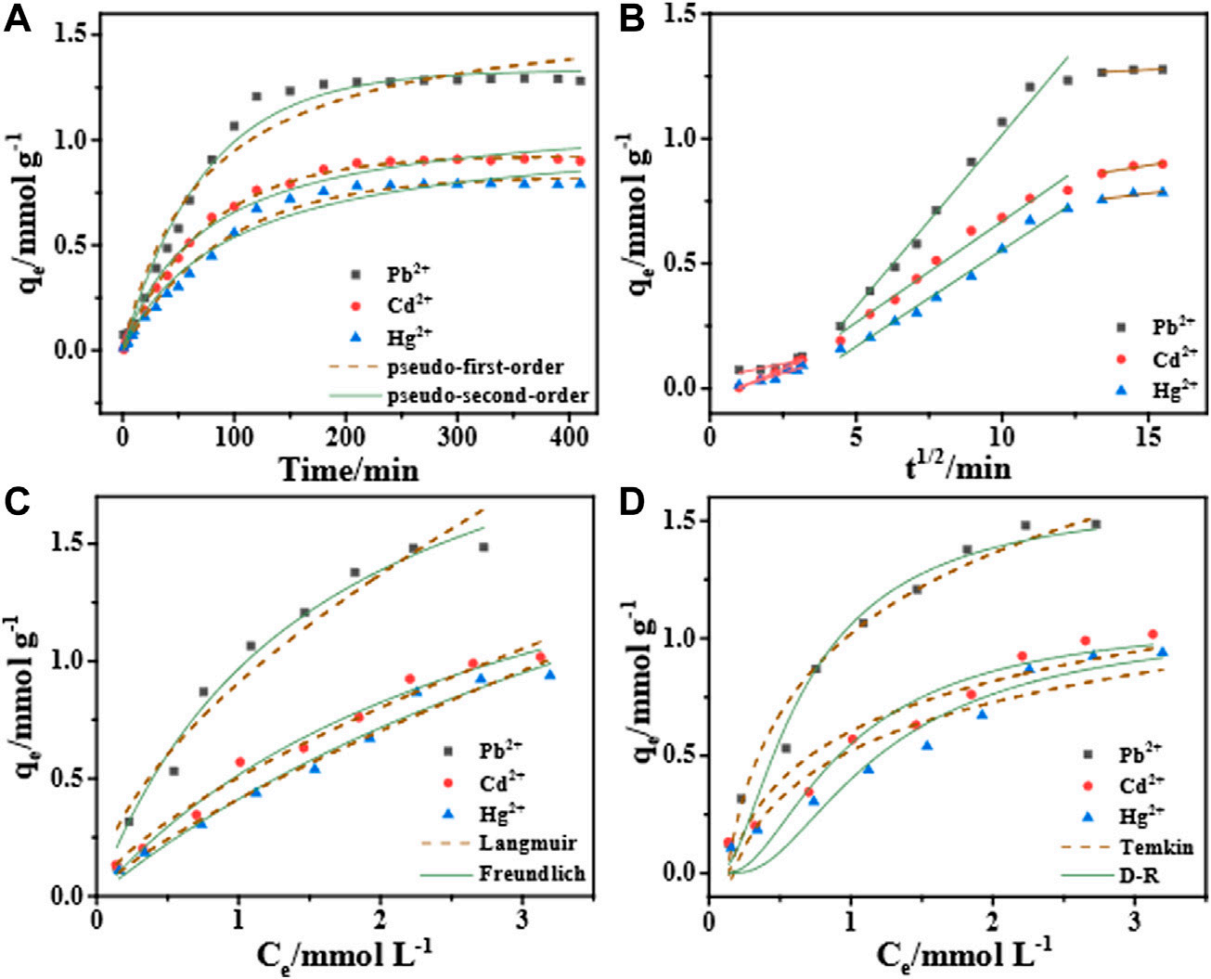
Adsorption kinetics of STS-PAA for Pb2^+^, Cd2^+^ and Hg2^+^: pseudo-first-order kinetics, pseudo-second-order kinetics **(A)**, intra-particle diffusion **(B)**; Adsorption isotherm: Langmuir, Freundlich **(C)**, Dubinin-Radushkevich, Temkin **(D)**.

Figure 8A shows that the change curve of adsorption capacity and time. The adsorption amount that the magnitude of the adsorption first increases rapidly with time and then tends to level off. The slope of the curve decreases with time. Further, the adsorption curves of each particle of Pb^2+^, Cd^2+^ and Hg^2+^ are fitted by the pseudo-first-order kinetic model, pseudo-second-order kinetic model and intra-particle diffusion model to further explain the mechanism of heavy metal particle adsorption by hydrogel. It can be seen from the Table 2 that the correlation coefficient *R* ^2^ value of the pseudo-first-order kinetic model is closer to 1.00. Therefore, the adsorption processes of STS-PAA for Pb^2+^, Cd^2+^ and Hg^2+^ are more consistent with the pseudo-first-order kinetic model. This indicates that the adsorption process of heavy metal ions by STS-PAA is mainly physical adsorption.

**TABLE 2:** The adsorption kinetic model parameters of STS-PAA.

Type of pollutant	Pb^2+^	Cd^2+^	Hg^2+^
Pseudo-first-order model			
q_e_ (mmol·g^−1^)	1.332	0.925	0.828
k_1_ (min^−1^)	0.014	0.013	0.011
*R* ^2^	0.988	0.998	0.991
Pseudo-second-order model			
q_e_ (mmol·g^−1^)	1.633	1.131	1.050
k_2_ (min^−1^)	0.060	0.020	0.012
*R* ^2^	0.972	0.990	0.980
Intraparticle diffusion linear fitting			
K_p1_(mmol g^−1^ min^−1/2^)	0.024	0.051	0.036
C_1_	0.038	−0.05	−0.029
R_1_^2^	0.593	0.984	0.877
K_p2_(mmol g^−1^ min^−1/2^)	0.138	0.081	0.077
C_2_	−0.364	−0.141	−0.218
R_2_^2^	0.981	0.975	0.984
K_p3_(mmol g^−1^ min^−1/2^)	0.006	0.019	0.014
C_3_	1.190	0.615	0.567
R_3_^2^	0.688	0.803	0.689

The intra-particle diffusion model in Figure 8B shows a three-stage linear relationship, which indicates that the adsorption process is controlled by three adsorption stages. The linear fit between the intra-particle diffusion model q and t^1/2^ does not pass through the origin, indicating that internal diffusion is not the only step in manipulating the adsorption process. In the first period, q has a poor linear relationship with t^1/2^. The linear relationship is good in the second period, which indicates that this stage is controlled by intra-particle diffusion in the adsorbent pores. The last stage is the final equilibrium stage when the diffusion rate in the particle slows down.

#### 3.4.2 Study on adsorption isotherm

Figures 8C,D is the fitting diagrams of the four isotherm models of the STS-PAA adsorbent for Pb^2+^, Cd^2+^ and Hg^2+^, respectively. At the same time, Table 3 is the relevant parameters of the four isotherm models. We can see through comparative analysis that the *R* ^2^ of Langmuir isotherm model is closer to 1, and higher than that of Freundlich isotherm model and Temkin isotherm model. This indicates that the adsorption sites of the STS-PAA adsorbent are uniformly distributed, and the adsorption manner is a single-layer physical adsorption.

**TABLE 3:** Isotherm model parameters of STS-PAA.

Type of pollutant	Pb^2+^	Cd^2+^	Hg^2+^
Langmuir			
K_L_ (L∙mmol^−1^)	0.230	0.179	0.011
q_m,l_ (mmol·g^−1^)	3.258	2.521	3.404
*R* ^2^	0.989	0.990	0.985
Freundlich			
K_F_ (L∙mmol^−1^)	0.601	0.386	0.321
n	1.407	1.353	1.221
*R* ^2^	0.972	0.985	0.984
Temkin			
A (mmol∙L^−1^)	3.880	4.043	3.734
B	0.533	0.343	0.321
*R* ^2^	0.958	0.922	0.886
D-R			
q_m,D-R_ (mmol·g^−1^)	1.616	1.094	1.054
β (mol^2^KJ^−2^) × 10^−3^	3.533	3.973	5.110
*R* ^2^	0.960	0.924	0.911

#### 3.4.3 Study on thermodynamic study

**FIGURE 9:**
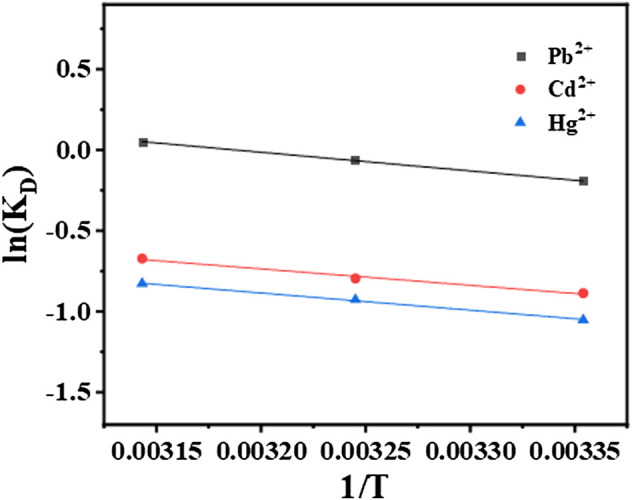
Adsorption thermodynamic of STS-PAA for Pb^2+^, Cd^2+^ and Hg^2+^.

The adsorption thermodynamic of STS-PAA for Pb2^+^, Cd2^+^ and Hg2^+^ are shown in Figure 9, and the thermodynamic parameters are shown in Table 4 and Table 5. ΔH refers to the enthalpy change between reactants and reaction products. In the process of adsorption of these three metal ions, the ΔH value is always positive, between 8.836 and 9.670kJ mol-1, which indicates that the essence of the ion adsorption process is endothermic reaction. Increasing the temperature is conducive to the adsorption of these three metal ions on STS-PAA. The values of ΔS are all positive, indicating that during the adsorption process, the disorder of solid-liquid surface increases, and the disorder of Pb^2+^ adsorption is significantly higher than that of Cd^2+^and Hg^2+^. ΔG is Gibbs free energy and is small and positive except for Pb^2+^ adsorption at 318.15K. This indicates that STS-PAA requires a small amount of energy to convert the reactants into products when adsorbing Pb^2+^, Cd^2+^and Hg^2+^. ΔG is a positive value, which is very common in the adsorption system (Unuabonah et al., 2008; Lu et al., 2016; Choi et al., 2017; You et al., 2018; Yang et al., 2022). Using the linear graph of the thermodynamic model to estimate the thermodynamic parameters may introduce some errors, which may transfer the values from one extreme boundary to another. When this happens, small and negative ΔG may become small and positive ΔG. (Unuabonah et al., 2008). Therefore, it does not mean that the adsorption of Pb^2+^, Cd^2+^and Hg^2+^ on the hydrogel is nonspontaneous. On the contrary, as the temperature of aqueous solution increases, ΔG tends to be negative, and the adsorbents show better adsorption performance for Pb^2+^, Cd^2+^ and Hg^2+^. The ΔH value is positive, which further supports this conclusion (Table 4).

**TABLE 4:** The adsorption thermodynamics of STS-PAA.

	ΔH (kJ mol^−1^)	ΔS (J mol^−1^ K^−1^)	*R* ^2^
Pb^2+^	9.670	30.623	0.999
Cd^2+^	8.449	20.916	0.989
Hg^2+^	8.836	20.915	0.998

**TABLE 5:** The adsorption Gibbs free energy of STS-PAA.

	T(K)	ΔG (kJ mol^−1^)
Pb^2+^	298.15	0.540
	308.15	0.234
	318.15	−0.073
Cd^2+^	298.15	2.213
	308.15	2.004
	318.15	1.795
Hg^2+^	298.15	2.600
	308.15	2.391
	318.15	2.182

## 4 Conclusion

In this study, a new tobacco straw-based polymer STS-PAA was synthesized by UV radiation initiation. The surface of this hydrogel can be observed by scanning electron microscopy as rough and pore-like structure, which can be used to adsorb heavy metal ions. The adsorption of Pb2+, Cd2+ and Hg2+ metal ions was systematically investigated at different pH, temperature, time, and ion concentrations. It turns out that the STS-PAA adsorbent has good adsorption capacity for Pb^2+^, Cd^2+^ and Hg^2+^ ions. The adsorption of Pb2+ ions was particularly effective, with the adsorption amount reaching 1.49 mmol g-1. The successful adsorption of metal ions could be visualized by SEM, EDS and XPS. The mechanism was analyzed by XPS and it was found that the interaction between -COO- and metal ions was the main factor. The pseudo-first-order kinetic model and Freundlich isotherm model can be well adapted to the adsorption data. This indicates that STS-PAA adsorption of three heavy metal ions is a physical monolayer adsorption. This paper provides some ideas and insights for the preparation of new environmentally friendly renewable hydrogels.

The main advantages of STS-PAA can be summed up in a few words. Raw materials are cheap, readily available, and natural. Tobacco straw is rich in lignin and cellulose, which is an ideal raw material for preparation of hydrogel materials. The synthesized polymer material has strong adsorption capacity and is friendly to environment. However, STS only showed strong adsorption capacity for Pb^2+^, while the adsorption capacity for Cd^2+^ and Hg^2+^ remained to be improved. In the future, we will use STS-PAA for remediation of sewage, study the adsorption of STS-PAA under the state of ion competitive adsorption, and recover the adsorbed STS-PAA material for repeatability study, to judge its application prospect.

## Data Availability

The raw data supporting the conclusion of this article will be made available by the authors, without undue reservation.
